# Primary Perinephric Abscess Caused by Mycobacterium abscessus in a Renal Transplant Recipient

**DOI:** 10.7759/cureus.108428

**Published:** 2026-05-07

**Authors:** Jyoti Prakash Panda, Mala Kaneria

**Affiliations:** 1 Microbiology, Jaslok Hospital & Research Centre, Mumbai, IND; 2 Infectious Diseases, Jaslok Hospital & Research Centre, Mumbai, IND

**Keywords:** drug-related side effects and adverse reactions, mycobacterium abscessus (m. abscessus), non-tuberculous mycobacteria, perinephric abscess, post-renal transplant

## Abstract

Non-tuberculous mycobacteria are increasingly recognized as opportunistic pathogens in solid organ transplant recipients, most commonly causing cutaneous, pulmonary, or disseminated disease. Primary perinephric involvement without evidence of dissemination is exceptionally rare. We report the case of a 34‑year‑old male renal transplant recipient who presented eight months post‑transplant with right iliac fossa pain and fever following minor trauma. Imaging revealed a perinephric collection initially suspected to be a hematoma. Routine bacterial cultures were sterile, and procalcitonin levels were low. Acid‑fast bacilli were detected on the smear, and mycobacterial culture identified *Mycobacterium abscessus*, a rapidly growing non‑tuberculous mycobacterium. The patient was successfully managed with percutaneous drainage and a tailored multidrug antimicrobial regimen, avoiding aminoglycosides to preserve graft function, although management was complicated by significant drug‑related toxicities and immunosuppressant interactions. This case highlights the importance of considering non‑tuberculous mycobacterial infection in culture‑negative perinephric collections in immunocompromised hosts and underscores the diagnostic and therapeutic challenges posed by *Mycobacterium abscessus* in renal transplant recipients.

## Introduction

Infections remain a significant cause of morbidity and mortality in kidney transplant recipients. While bacterial and viral pathogens are common [1], non‑tuberculous mycobacteria are increasingly recognized as opportunistic pathogens in this population, with an estimated incidence ranging from 0.16% to 0.55% [2,3]. Among non‑tuberculous mycobacterial species, *Mycobacterium abscessus*, a rapid‑growing mycobacterium, poses a particular therapeutic challenge owing to its intrinsic resistance to many anti‑tubercular drugs and broad‑spectrum antibiotics [4].

Non‑tuberculous mycobacterial infections in solid organ transplant recipients typically manifest as cutaneous or soft tissue infections, pulmonary disease, or disseminated infection [5]. Primary involvement of the renal graft or perinephric space is uncommon and is most often associated with early postoperative surgical site infections [6]. We describe a rare case of primary perinephric *Mycobacterium abscessus* infection presenting eight months after renal transplantation, likely resulting from secondary infection of a traumatic hematoma, and discuss the associated diagnostic and therapeutic challenges.

## Case presentation

A 34‑year‑old male physician, a recipient of a living‑donor renal transplant from his mother in October 2022 for end‑stage renal disease secondary to IgA nephropathy, presented to our center in June 2023. He was receiving maintenance immunosuppression with tacrolimus (4.5 mg/day), mycophenolate mofetil (1,000 mg/day), and prednisolone (5 mg/day). His baseline serum creatinine prior to presentation was stable at 1.2 mg/dL.

The patient reported a history of trivial trauma during exercise on May fifteen, 2023, following which he developed persistent tenderness over the right iliac fossa corresponding to the graft site. A computed tomography scan performed externally on May 19, 2023, demonstrated mild swelling of the transplant kidney with a mixed-density collection in the perinephric space, which was interpreted as a hematoma. Approximately two weeks later, he developed intermittent high‑grade fever with a maximum recorded temperature of 101°F, while the local pain persisted. He denied respiratory, gastrointestinal, or urinary symptoms.

On admission, the patient was hemodynamically stable. Physical examination revealed localized tenderness in the right iliac fossa without palpable organomegaly or graft bruit. The surgical incision was well healed with no evidence of superficial wound infection.

Laboratory investigations showed leukocytosis, with a total leukocyte count of 15,500 cells/mm³, and elevated inflammatory markers, including a C‑reactive protein (CRP) level of 30.30 mg/dL and an erythrocyte sedimentation rate of 34 mm/hour. Procalcitonin was low at 0.2 ng/mL. Renal function had worsened, with serum creatinine rising to 1.84 mg/dL. Urinalysis revealed one to two pus cells per high‑power field.

Computed tomography of the pelvis performed on June 8, 2023, revealed a hypointense collection measuring 5.2 × 6.2 × 9.0 cm (approximately 120 cc) in the perinephric region surrounding the transplanted kidney (Figure 1). Positron emission tomography-computed tomography demonstrated multiple thick‑walled metabolically active collections around the graft, with no evidence of pulmonary or disseminated disease.

**Figure 1:**
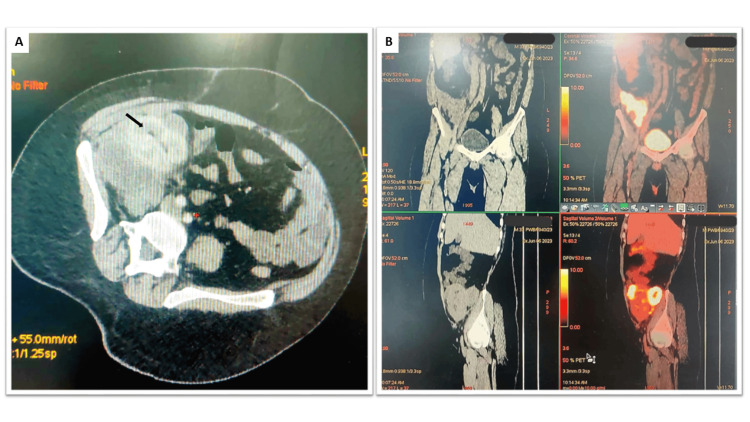
Radiological assessment of the perinephric collection (A) Axial computed tomography scan of the pelvis demonstrating a hypodense collection (black arrow) in the right perinephric space, initially mimicking a hematoma. (B) Positron emission tomography-computed tomography scan demonstrating intense peripheral fludeoxyglucose-uptake around the transplant kidney, confirming an active infectious/inflammatory collection.

Ultrasound‑guided aspiration of the collection yielded reddish, hazy pus with markedly elevated lactate dehydrogenase (14,794 U/L) and adenosine deaminase levels (341 U/L). Gram staining revealed abundant pus cells without identifiable organisms. Ziehl‑Neelsen staining demonstrated pleomorphic acid‑fast bacilli, some arranged in cuneiform patterns.

Cartridge‑based nucleic acid amplification testing (CB NAAT) for *Mycobacterium tuberculosis* was negative. A non‑tuberculous mycobacterial polymerase chain reaction (NTM-PCR) performed at a reference laboratory was positive but failed to identify the species. Routine bacterial and fungal cultures remained sterile. Mycobacterial culture using a BD BACTEC Myco/F lytic system (Becton, Dickinson and Company, Franklin Lakes, NJ, USA) flagged positive after four days, suggestive of a rapid‑growing mycobacterium, which was subsequently isolated on Lowenstein-Jensen (LJ) culture medium and identified as *Mycobacterium abscessus* using matrix‑assisted laser desorption/ionization time‑of‑flight mass spectrometry (Figure 2).

**Figure 2:**
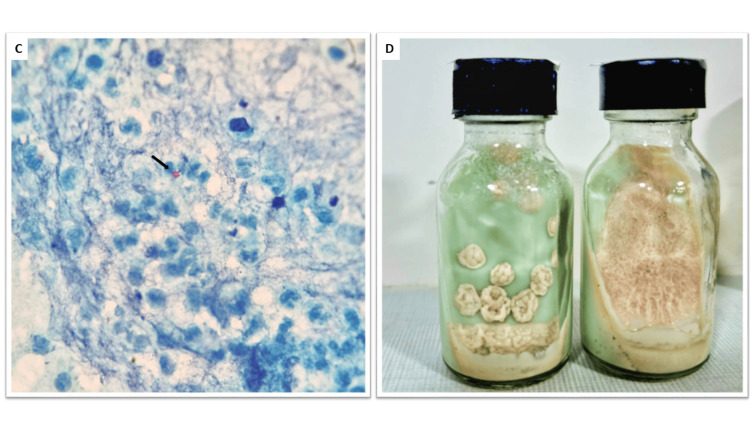
Microbiological identification of Mycobacterium abscessus (C) Ziehl-Neelsen stain of the ultrasound-guided aspirate revealing a solitary acid-fast bacillus (black arrow) amidst a background of numerous pus cells. (D) Lowenstein-Jensen culture medium showing the rapid growth of characteristic cream colored, rough, and friable colonies.

A pigtail catheter was inserted for source control. Based on smear positivity and negative CB NAAT testing for *Mycobacterium tuberculosis*, the patient was empirically initiated on intravenous imipenem, linezolid, clarithromycin, and moxifloxacin while awaiting culture identification and drug susceptibility results. Amikacin, although a preferred agent for rapidly growing mycobacterial infections, was withheld to minimize the risk of nephrotoxicity and preserve graft function.

The patient developed severe nausea and vomiting attributed to imipenem, necessitating substitution with intravenous tigecycline administered as a 100 mg loading dose followed by 50 mg twice daily. Drug susceptibility testing subsequently demonstrated resistance to moxifloxacin, leading to modification of therapy to tigecycline, clarithromycin, and linezolid. Linezolid was initiated with careful hematological monitoring. Retrospective review revealed concurrent selective serotonin reuptake inhibitor therapy for anxiety, prompting increased vigilance for serotonin syndrome. After three weeks of therapy, the total leukocyte count declined to 4,060 cells/mm³, and linezolid was discontinued due to drug‑induced myelosuppression. Long‑term parenteral therapy with tigecycline was considered impractical, and the regimen was transitioned to oral clofazimine at a dose of 100 mg once daily. Four weeks into therapy, tacrolimus trough levels increased to 14.2 ng/mL, necessitating temporary withholding of clarithromycin.

The patient became afebrile within 72 hours of percutaneous drainage and appropriate antimicrobial therapy. Serial ultrasonography demonstrated progressive resolution of the abscess cavities. By August 2023, approximately two months after discharge, the collection had reduced to less than one cubic centimeter. Renal function stabilized with a serum creatinine of 1.38 mg/dL, and inflammatory markers normalized. The collection continued to regress on subsequent imaging, and a total of six months of antimicrobial therapy was completed.

## Discussion

Non‑tuberculous mycobacteria are ubiquitous environmental organisms commonly found in soil and water [6]. Although pulmonary disease is the most frequent manifestation in the general population [7], solid organ transplant recipients are particularly susceptible to extrapulmonary and disseminated infections owing to impaired cell‑mediated immunity related to long‑term T‑cell-directed immunosuppression [8].

Pathogenesis and clinical presentation

*Mycobacterium abscessus* is a rapidly growing, multidrug‑resistant non‑tuberculous mycobacterium that poses unique clinical challenges. In renal transplant recipients, non‑tuberculous mycobacterial infections typically arise either from reactivation of a latent infection or from direct inoculation through environmental or nosocomial exposure [9]. Most reported cases involve surgical site or soft tissue infections occurring within the first few months following transplantation [10]. In contrast, our patient presented eight months after transplant, making early postoperative contamination less likely.

The history of minor trauma in our case suggests that the resulting perinephric hematoma may have acted as a locus minoris resistentiae, representing a localized area of reduced tissue resistance. This compromised microenvironment likely facilitated hematogenous seeding during a transient episode of mycobacteremia, presumably originating from an environmental source, most plausibly contaminated water, ultimately leading to abscess formation [11].

Diagnostic challenges

The diagnosis of non-tuberculous mycobacterial infection is frequently delayed, as clinical features often mimic bacterial abscesses or graft dysfunction, including acute rejection. A key diagnostic clue was the 'sterile' routine culture despite abundant pus cells, paired with a disproportionately low procalcitonin (0.2 ng/mL), below the accepted bacterial infection threshold of 0.5 ng/mL [12]. This is consistent with the known tendency of mycobacterial infections to produce lower procalcitonin responses compared with typical bacterial sepsis. [13]. The CRP level in our patient, though elevated at 30.30 mg/dL, was modest relative to values typically observed in acute bacterial sepsis. This attenuated acute-phase response likely reflects the blunted inflammatory signaling inherent to chronic immunosuppressive therapy. Furthermore, the combination of a positive acid-fast bacilli smear with a negative CB-NAAT for Mycobacterium tuberculosis should immediately raise suspicion for non-tuberculous mycobacteria [14].

Therapeutic considerations

Management of *Mycobacterium abscessus* infection remains challenging because of its intrinsic resistance to standard anti‑tubercular drugs and multiple classes of broad‑spectrum antibiotics. Current recommendations favor a macrolide‑based multidrug regimen, guided by drug susceptibility testing whenever feasible [15]. In kidney transplant recipients, however, treatment is complicated by significant drug-drug interactions and toxicity concerns.

Macrolides, particularly clarithromycin, inhibit cytochrome P450 3A4, resulting in elevated calcineurin inhibitor levels and increasing the risk of tacrolimus toxicity, as observed in our patient [16]. Aminoglycosides such as amikacin are a cornerstone of therapy for rapidly growing mycobacterial infections but carry a substantial risk of nephrotoxicity, which is especially concerning in renal allograft recipients and may be potentiated by concurrent nephrotoxic immunosuppressive agents [17]. In this context, alternative agents such as tigecycline and clofazimine were successfully employed in our patient to limit further graft injury.

Treatment duration for *Mycobacterium abscessus* infections is typically prolonged, often extending six to 12 months, necessitating close clinical and laboratory monitoring. Long‑term therapy increases the risk of adverse events, including linezolid‑induced myelosuppression, which required discontinuation of the drug in our patient [18]. These challenges underscore the importance of individualized, closely monitored treatment strategies in transplant recipients with non‑tuberculous mycobacterial infections.

## Conclusions

Primary perinephric abscesses are most commonly caused by gram‑negative enteric organisms such as *Escherichia coli* and *Klebsiella pneumoniae*, typically arising secondary to recurrent urinary tract infections or pyelonephritis. In contrast, primary perinephric abscess due to non‑tuberculous mycobacteria is exceedingly rare but represents a potentially serious complication in renal transplant recipients.

This case highlights the importance of maintaining a high index of suspicion for atypical pathogens in immunocompromised patients presenting with culture‑negative perinephric collections, particularly in the presence of predisposing factors such as trauma or hematoma formation. Early source control, careful microbiological evaluation, and individualized antimicrobial therapy that minimizes nephrotoxicity, along with close clinical monitoring, can result in favorable patient and graft outcomes. As with all single-case observations, the findings of this report should be interpreted with appropriate caution, and conclusions regarding optimal diagnostic and therapeutic strategies cannot be broadly generalized. Accumulation of additional cases through multicenter collaborative studies will be essential to establish evidence-based guidelines for this rare but clinically significant entity.
